# A Clinico-Etiology and an Outcome Analysis of Surgical Management of Choledocholithiasis

**DOI:** 10.7759/cureus.92099

**Published:** 2025-09-11

**Authors:** Sreekar Amaravadi, Nag Naveen Challagundla

**Affiliations:** 1 General Surgery, Chalmeda Ananda Rao Institute of Medical Sciences, Bommakal, IND; 2 Surgery, Dr. Pinnamaneni Siddhartha Institute of Medical Sciences and Research Foundation, Chinna Avutapalli, IND

**Keywords:** choledochoduodenostomy, ercp, laparoscopic cbd exploration, roux-en-y hepaticojejunostomy, t-tube repair

## Abstract

Aim

To assess the postoperative outcomes of various surgical techniques for choledocholithiasis, such as open common bile duct (CBD) exploration, laparoscopic CBD exploration, and a combined endoscopic and surgical approach, with an emphasis on immediate morbidity and long-term results.

Methods

This non-randomized prospective observational study was conducted over two years, with a median follow-up of 12 months. The study included 50 patients diagnosed with CBD stones, and their demographic data, preoperative factors, interventions (endoscopic and surgical), and postoperative outcomes were analyzed.

Results

The mean age of the patients was 53.88±9.49 years and 27 (54%) patients were women. The primary symptom was abdominal pain (49, 98%), followed by jaundice (41, 82%) and fever (15, 30%). Complications related to CBD stones occurred in 15 (32%) cases, including acute cholangitis (11, 22%), gallstone pancreatitis (three, 6%), and choledochoduodenal fistula (two, 4%). Most stones were located in the mid-CBD in 22 (44%) cases and distal CBD in 20 (40%) cases. In terms of quantity, 29 (58%) had multiple stones, while 21 (42%) had one. Regarding the size, 16 (32%) stones were small, 20 (40%) were large, and three (6%) were very large. The mean CBD diameter was 15.09±5.99 mm, with most cases between 15 to 20 mm. The mean total bilirubin was 5.71 mg/dl, direct bilirubin 4.51 mg/dl, alkaline phosphatase 542.50 IU, and the international normalized ratio (INR) was 1.30. A comparative analysis of endoscopic retrograde cholangiopancreatography (ERCP) combined with laparoscopic cholecystectomy (LC) and laparoscopic CBD exploration shows both methods produce similar short-term results (stone clearance, postoperative morbidity, hospital stay) and long-term outcomes (stone recurrence, strictures). However, the Laparoscopic Common Bile Duct Exploration (LCBDE) group had a significantly longer operative time, averaging 328±19.24 minutes, compared to 149.09±17.58 minutes for ERCP+LC. Long-term outcomes were also good, with a stricture and stone recurrence rate of 2.94%. A comparison of biliary drainage methods post-open CBD exploration (choledochoduodenostomy, Roux-en-Y hepaticojejunostomy, T-tube repair) showed similar short-term outcomes. The mean operative time for Roux-en-Y was 215±22.76 minutes, longer than choledochoduodenostomy (167.14±22.68 minutes) or T-tube repair (167.50±16.69 minutes).

Conclusion

Laparoscopic management of CBD stones requires significant expertise and has a steep learning curve. Our study shows that open CBD exploration is effective for complex CBD stone cases, especially after failed ERCP, despite higher short-term morbidity.

## Introduction

The prevalence of common bile duct (CBD) stones in patients with symptomatic gallstones is between 10% and 20%. However, in those without clinical signs of CBD stones before surgery, the incidence drops to under 5% [1]. Between 2% and 4% of gallstone patients may develop symptoms within a year. CBD stones can often be asymptomatic but may cause serious complications like complete obstruction and jaundice [2]. Unlike gallstones, the progression of CBD stones is less understood. Complications can include pain, obstructive jaundice, cholangitis, hepatic abscesses, pancreatitis, and secondary biliary cirrhosis, though not all patients will experience these issues. Some may even expel CBD stones into the duodenum before or after laparoscopic cholecystectomy. There is no specified preferred technique for CBD stone extraction; the choice depends on the patient's condition, the surgeon's expertise, institutional protocols, and available resources. Numerous studies, including randomized trials and meta-analyses, have been conducted, but results are inconsistent [3].

Management strategies for CBD stones include a two-stage method combining ERCP with laparoscopic cholecystectomy (LC) and a one-stage procedure involving either laparoscopic or open cholecystectomy with CBD exploration and ERCP, which is the preferred approach. In many developing countries, surgeons often perform single-stage open cholecystectomy with CBD exploration due to limited skills in minimally invasive techniques like ERCP and laparoscopy. Cholecystocholedocholithiasis and its complications are frequently seen in surgical outpatient departments and emergency rooms [4]. Although the two-stage method is widely used, it has drawbacks, such as the risk of stones passing before ERCP, CBD stones moving between ERCP and LC, and the need for multiple anesthesia sessions and hospital stays. Given the lack of consensus on the best treatment for this common issue, this study aims to share our institutional experience.

This non-randomized prospective study involved 50 cases of CBD stones treated through various methods (open CBD exploration, laparoscopic CBD exploration, and a combined endoscopic and surgical approach).

This study assesses the postoperative outcomes of various surgical techniques for choledocholithiasis, including open CBD exploration, laparoscopic CBD exploration, and a combined endoscopic and surgical approach, with an emphasis on immediate morbidity and long-term results.

## Materials and methods

A prospective, non-randomised, and observational study was carried out in the Department of Surgery. A total of 50 cases were admitted to the Department of Surgery.

The sample size is calculated based on the following formula: 

$n = \frac{Z^2 \cdot p \cdot q}{d^2}$

where n=sample size; Z=1.96 ≈ 2 (considering confidence as 95%); p=prevalence (considered as 14% as exact prevalence is not known); q=100-p, that is, 86%; d=absolute error, which was 10%.

$n = \frac{2 \times 2 \times 0.14 \times 0.86}{0.1 \times 0.1}$

which makes it n=48.16.

The calculated sample size is 48.16 and hence we have taken sample size of 50.

Case definition

Patients with right upper quadrant and epigastric pain, with or without jaundice, fever, vomiting, or dyspepsia, were evaluated. Each patient underwent an abdominal ultrasound and liver function tests (LFT). Those suspected of having CBD stones based on ultrasound findings (gallstones and enlarged CBD) were referred for magnetic resonance cholangiopancreatography (MRCP) for the confirmation of diagnosis.

All diagnosed cases of CBD stones with an age greater than 12 years, patients with CBD stones who had a failed attempt at therapeutic ERCP, CBD stones presenting as acute cholangitis, gallstone pancreatitis, and choledochoduodenal fistula, and CBD stones coexisting with acute cholecystitis, Mirizzi syndrome, or distal CBD stricture were included.

Whereas patients with gallstones without any predictors of choledocholithiasis, patients with severe gallstone pancreatitis and grade III acute cholangitis, cases of CBD stones less than 12 years, American Society of Anesthesiology (ASA) class 4 and 5 disease, patients who refused to give consent, and pregnancy cases were excluded.

Study protocol

After obtaining informed consent, study participants underwent examinations using a pretested proforma, followed by various interventional procedures. A thorough physical examination and preoperative data was collected. Laboratory investigations were performed, such as complete blood count, liver function tests, serum lipase and amylase, blood sugar, blood urea, serum creatinine, and coagulation profile. All patients then had an abdominal ultrasound, with further imaging (MRCP, contrast-enhanced computed tomography (CECT), or endoscopic ultrasonography (EUS)).

The patients (n=50) were subsequently subjected to intervention as follows [5]: in Intervention A, ERCP followed by laparoscopic cholecystectomy; in Intervention B, laparoscopic cholecystectomy with CBD exploration; and in Intervention C, open cholecystectomy with CBD exploration were performed.

CBD exploration in both laparoscopy and open cholecystectomy was followed by either T-tube repair or bilioenteric anastomosis. The decision to use a drainage procedure (T-tube or bilioenteric anastomosis) during open or laparoscopic common bile duct exploration (CBDE) was based on factors such as previous endoscopic attempts, presence of strictures, history of recurrent cholangitis, duct diameter, stone quantity, and past upper abdominal surgeries.

Intervention 1: ERCP followed by laparoscopic cholecystectomy

Under sedation or general anaesthesia, a side-viewing endoscope is inserted into the duodenum's second segment to visualize the ampulla. The major papilla is cannulated with a guidewire using fluoroscopic guidance. After exchanging for a sphincterotome, it is retracted until one-fourth to one-third of the cutting wire remains in the papilla. The sphincterotome's tip is bent to ensure the cutting wire contacts the papilla's roof and is directed towards the biliary sphincter at the 11 to 1 o’clock position, with no more than 5 mm of wire inside. Endoscopic sphincterotomy (EST) can extend along the bile duct axis to the duodenal wall. A cholangiogram confirms the stone's location in the CBD. Standard retrieval tools include balloon catheters, Dormia baskets, and mechanical lithotripters. A balloon catheter is advanced over the guidewire into the CBD, inflated, and retracted to remove the stone or sludge. If unsuccessful, a Dormia basket is used under fluoroscopic guidance to retrieve the stone. After extraction, a balloon catheter sweep of the CBD is performed to clear any residual sludge or small stones.

Mechanical lithotripsy is the simplest method for breaking down CBD stones larger than 1.5 cm. It involves using a basket within a metal sheath, guided over a wire to access the CBD. The sheath is then removed, trapping the stone in the basket, which is crushed against a metal spiral sheath to reduce the stone's size for easier retrieval. A balloon sweep is also done to clear any sludge or leftover stone. A 7 Fr-10 Fr plastic stent is placed in the CBD. Subsequently, a standard four-port laparoscopic cholecystectomy is performed within 24-48 hours after ERCP.

Upfront surgical procedures

The principles for CBD exploration with choledochoduodenostomy (CD) or hepaticojejunostomy (HJ) were consistent in both traditional and laparoscopic surgeries, aiming for a broad, diamond-shaped anastomosis. All patients who had CD/HJ underwent a side-to-side choledochoduodenostomy, ideally following Gliedman and Gold's original method [6].

Intervention 2: laparoscopic CBD exploration

Patients underwent laparoscopic exploration of CBD using a four-port method with CO_2_ pneumoperitoneum at 14 mmHg and 8 L/min flow, employing the open Hasson technique. A 10 or 12 mm trocar was placed at the umbilicus for the camera, and a 5 or 10 mm trocar was positioned in the sub-xiphisternum as the main working port. Two 5 mm trocars were inserted in the right upper quadrant, 2 cm below the costal margin along the anterior axillary and mid-clavicular lines. A 30° angled video-laparoscope was introduced through the umbilical port. After diagnostic laparoscopy, careful dissection was performed to release adhesions until the duodenum and portal triad were visible. Calot's anatomy was defined, and the cystic artery was secured with LT 300 titanium clips before transection. The cystic duct was clipped with an LT 300/400 clip toward the gallbladder (GB) and divided, keeping the GB attached to the hepatic bed for upward traction to expose the infrahepatic region. A generous Kocher's maneuver was performed for a tension-free anastomosis. The CBD was incised longitudinally with a monopolar hook, starting where it crosses the duodenum and extending proximally for about 2.5 cm. Stone extraction was mainly done through milking, aided by saline irrigation with an infant feeding tube.

Any previously inserted stent is removed. The proximal and distal ducts are irrigated with warm saline to clear debris and infected fluids. Choledochoscopy is performed through a 5-mm right subcostal port using a rigid nephroscope, or an additional port may be added. If stone clearance is incomplete, stones are identified and extracted using endoscopic tools like baskets and balloons, or the procedure may convert to open surgery. The duodenum is incised longitudinally along its superior margin for about 1.5 cm. A single-layer anastomosis is performed with 4-0 Vicryl/PDS (polydioxanone) interrupted sutures. After the anastomosis, the gallbladder is detached from the liver bed and removed in an endo bag. A closed drain is placed laterally to the anastomosis, directed towards the Hepatorenal/Morrison’s space, and the fascia and skin are approximated.

Intervention 3: open CBD exploration

The procedure begins with a right subcostal incision. The duodenum is extensively kocherized before performing a choledochotomy, allowing for palpation of the distal common duct as it passes behind the duodenum and pancreas before entering the second segment of the duodenum. The common duct is cleaned over a 2-3 cm length, typically between the cystic duct stump and the duodenum. Stay sutures of 5-0 synthetic non-absorbable material are placed on the common duct, followed by a choledochotomy of at least 1.5 cm to allow for easy manipulation without trauma. Stones may be expelled as bile flows from the duct, and any detected stones can be moved toward the choledochotomy for removal. A 12 Fr catheter is introduced through the choledochotomy, extending into the hepatic ducts, and is irrigated with saline while being gradually retracted to clear sludge and small stones. Desjardin’s forceps are used to remove calculi from the biliary tree, ensuring the choledochotomy is adequately sized to prevent trauma. After clearing the biliary tract, it is irrigated again with saline. An intraoperative cholangiogram is performed, followed by either a T-tube closure or a bilioenteric anastomosis (CD or HJ).

T-tube closure

A 14F T-tube is typically used, inserted with Desjardin’s forceps. The ends and posterior section are trimmed for proper size and drainage. Closure of the choledochotomy starts at the top, positioning the T-tube at the bottom with 4-0 or 5-0 Vicryl sutures. A closing cholangiogram checks duct clearance. The T-tube exits laterally through the abdominal wall, and an abdominal drain is placed at the choledochotomy and T-tube closure site.

Choledochoduodenostomy

A longitudinal duodenotomy is performed, slightly smaller than the choledochotomy to allow for duodenal distensibility. Interrupted 4-0 or 5-0 PDS sutures are used to anastomose the posterior and anterior layers, connecting the duct to the duodenal mucosa. Three-corner 4-0 or 5-0 absorbable sutures are placed: two between the midpoints of the ductotomy and the duodenotomy ends, and one between the distal CBD incision and the duodenotomy's superior lip. Traction on these sutures reorients the ductotomy, aiding in the placement of posterior sutures between the duodenotomy's superior edge and the distal CBD. Full-thickness sutures connect the ductal and duodenal mucosa. A fourth corner suture may be added between the ductotomy's proximal end and the anterior duodenal wall midpoint, lifting the anterior wall for easier placement of remaining sutures [7].

Roux-en-Y hepaticojejunostomy

The procedure can be performed as either an end-to-side or side-to-side anastomosis. A 50 to 70 cm Roux-en-Y jejunal limb is positioned retrocolic, adjacent to the proximal bile duct without tension. Anterior full-thickness interrupted sutures (4-0 or 5-0 PDS) are placed from exterior to interior, followed by a posterior row to connect the duct and jejunum. The posterior sutures are secured internally, and the anterior sutures finalize the anastomosis with knots tied externally. Patients were monitored for up to 12 hours in a high-dependency unit and followed up at six weeks, three months, six months, and one year, or sooner if symptoms occurred, through clinical exams, liver function tests, ultrasounds, and assessments for CBD clearance and potential complications [8].

The primary goal was to clear stones from the common bile duct, confirmed by intraoperative cholangiogram, and to lower bilirubin levels and conduct necessary radiological assessments post-surgery. Secondary objectives included short-term outcomes like immediate postoperative morbidity, mortality, pain scores (Visual Analog Score (VAS)), hospital stay duration, and recovery time, as well as long-term outcomes such as stone recurrence, bile duct strictures, and incisional hernia.

Ethical statement

The study was conducted after obtaining approval from the institutional ethical committee, Nimra Institute of Medical Sciences (NIMS) (IEC/VJA/2023.01 dated April 15, 2023). Informed consent was obtained from the patients before their enrolment in the study.

Statistical analysis

Qualitative variables were shown as percentages, while quantitative variables were categorized and expressed as percentages or means with standard deviations. Data was analysed by using software Statistical Package for Social Studies (SPSS 26; IBM Corp., Armonk, NY, USA). Student’s t-test was applied to analyze the difference between the mean levels of various parameters between the groups. Categorical data were tested using the chi-squared test. A p-value of <0.05 was considered statistically significant.

## Results

This study includes a total of 50 cases, with participants having an average age of 53.88±9.49 years. The symptom most frequently reported was abdominal pain, present in 98% of cases, followed by jaundice in 41 (82%) cases and fever in 15 (30%) cases. In the study, 12 (24%) participants experienced a combination of abdominal pain, fever, and jaundice. A notable 27 (54%) cases were linked to symptomatic gallstone disease. Additionally, three (6%) had distal CBD stricture, post-cholecystectomy in one case, and three (6%) had acute cholecystitis, with one case involving Mirizzi syndrome. Complications related to CBD stones were observed in 16 (32%) cases, including acute cholangitis in 11 (22%), gallstone pancreatitis in three (6%), and choledochoduodenal fistula in two (4%). The mid-CBD was the most common location for stones in 22 (44%) patients, followed by the distal CBD in 20 (40%) patients. The average CBD diameter was 15.09±5.99 mm, with most cases ranging from 15 to 20 mm. Furthermore, among the total 50 cases, 29 (58%) cases had multiple stones, while 21 (42%) had a single stone. The distribution of stone sizes was 16 (32%) small stones, 20 (40%) large stones, and three (6%) very large stones among total 50 cases (Tables 1, 2). Whereas among the cases of ERCP+LC group and LCBDE group, four cases had multiple stones, while 12 cases had a single stone and among the distribution of stone sizes, 11 cases had small stones, five cases had large stones, and no cases had very large stones (Table 1).

**Table 1:** Various factors on basis on the intervention endoscopic retrograde cholangiopancreatography (ERCP) + laparoscopic cholecystectomy (LC) and laparoscopic CBD exploration (LCBDE) (n=16) CBD: Common bile duct; VAS: Visual Analog Score; ASA: American Society of Anesthesiologists; SGOT: aspartate aminotransferase; SGPT: alanine aminotransferase; NPO: nil per os (or) nothing by mouth. .

Various factors	ERCP + LC		LCBDE		P value
	Number or Mean	Percentage (or) standard deviation	Number or Mean	Percentage (or) standard deviation	
Age	52.73	9.30	50.00	6.56	0.5
Gender					
Female	5	45.45	1	20.00	0.3
Male	6	54.55	4	80.00	
Comorbidity					
Present	8	72.73	1	20.00	0.04
Absent	3	27.27	4	80.00	
ASA					
Class 2	6	54.55	5	100.00	0.06
Class 3	5	45.45	0	0	
Site of stone					
Ampulla	3	27.27	0	0	0.07
Distal CBD	7	63.64	2	40.00	
Mid- and distal CBD	0	0	0	0	
Mid-CBD	1	9.09	3	60.00	
CBD diameter	7.41	5.52	16.60	2.70	0.003
Number of stones					
Multiple	2	18.18	2	40.00	0.3
Single	9	81.82	3	60.00	
Size of stones					
Small	10	90.91	1	20.00	0.004
Large	1	9.09	4	80.00	
Very Large	0	0	0	0	
Liver function tests					
Total serum bilirubin	4.78	2.27	5.02	0.76	0.8
Direct bilirubin	3.63	2.00	3.84	0.79	0.8
Alkaline phosphatase	474.09	135.35	553.20	71.20	0.2
SGOT	39.55	11.93	36.80	6.72	0.6
SGPT	42.91	16.96	45.60	15.58	0.7
Operative time	149.09	17.58	328.00	19.24	<0.001
Intra operative complications	1	9.09	0	0	0.4
Postoperative complications					
Post-operative ICU requirement	0	0	0	0	-
Bile leak	0	0	1	20	0.1
Bleeding	0	0	0	0	-
Incisional hernia	0	0	0	0	-
Pulmonary complications	0	0	0	0	-
Missed stones	0	0	0	0	-
Recurrent stones	1	9.09	1	20	0.5
Wound infections	0	0	0	0	-
Stricture	0	0	1	20	0.1
Post-ERCP pancreatitis, perforation, cholangitis, bleeding	0	0	0	0	-
Mortality	0	0	0	0	-
NPO status	0.18	0.40	2.60	1.52	0.0002
Duration of hospital stay	6.27	0.79	6.80	1.3	0.3
VAS at 24 hours	3.73	0.90	4.20	0.4	0.2
VAS at discharge	0.27	0.47	0.60	0.5	0.2
Time to return to work	14.09	1.87	16.20	3.3	0.1
Stone clearance rate	100%	-	100%	-	-

**Table 2:** Distribution of different factors in open cases (n=34) ERCP: endoscopic retrograde cholangiopancreatography; CBD: common bile duct; VAS: Visual Analog Score; SGOT: aspartate aminotransferase; SGPT: alanine aminotransferase; NPO: nil per os (or) nothing by mouth; INR: international normalised ratio.

Different factors	Open CBD exploration	
	Number or Mean	percentage (or) standard deviation
Age	54.82	9.9
Gender		
Female	21	61.7
Male	13	38.2
Comorbidity		
Present	18	52.9
Absent	16	47.05
Failed ERCP		
Yes	9	100
No	25	73.5
Presentation		
Abdominal pain	34	100
Jaundice	30	88.2
Fever	11	32.3
Abdominal pain plus jaundice	30	88.2
Abdominal pain plus fever	11	32.3
Abdominal pain plus jaundice plus fever	8	23.5
Site of stone		
Ampulla	0	0
Distal CBD	11	32.5
Mid- and Distal CBD	5	14.7
Mid-CBD	18	52.9
CBD diameter	17.3	4.2
Number of stones		
Multiple	25	73.53
Single	9	26.47
Size of stones		
Small	15	44.12
Large	16	47.06
Very Large	3	8.82
Basic blood investigations		
White blood cell count	10961.7	2960.1
Platelets (in lakh)	1.87	0.59
Serum creatinine	1.10	0.15
Liver function tests		
Total serum Bilirubin	6.11	1.44
Direct Bilirubin	4.89	1.29
Alkaline Phosphatase	563.06	107.6
SGOT	44.59	14.72
SGPT	46.41	19.08
Serum albumin	3.29	0.24
INR	1.29	0.23
Operative time	184.1	31.1
Intraoperative complications	0	0
Postoperative complications		
Postoperative ICU requirement	2	5.88
Bile leak	3	8.82
Incisional hernia	2	5.88
Pulmonary complications	11	32.3
Recurrent stones	1	2.94
Wound infections	10	29.4
Stricture	1	2.9
Mortality	0	0
NPO status	2.68	0.7
Duration of hospital stay	6.97	1.1
VAS at 24 hours	5.44	0.7
VAS at discharge	1.15	0.6
Time to return to work	18.76	2.6

Eleven (22%) patients underwent ERCP alongside laparoscopic cholecystectomy, while five (10%) had laparoscopic CBD exploration, and 34 (68%) underwent open CBD exploration. In the 39 (78%) cases that involved both open and laparoscopic techniques, 17 (43.59%) were treated with choledochoduodenostomy, 12 (30.77%) with Roux-en-Y hepaticojejunostomy, and 10 (25.64%) with T-tube repair. The average surgical time was 190.8 minutes, ranging from 120 to 350 minutes. The most common postoperative complications were pulmonary complications in 11 (22%) followed by wound infections in 10 (20%) cases. There was no mortality in this case series. The mean hospital stay was 6.8 days with a range of five to nine days.

Among the cases of open CBD exploration, the most common postoperative complication were pulmonary complications in 10 cases, followed by wound infections in 10 cases. There was no mortality in this case series (Table 2). 

Among the cases that underwent choledochoduodenostomy, there was no mortality. Similarly the common postoperative complication is pulmonary complications, and wound infections without mortality observed in cases underwent Roux-en-y hepaticojejunostomy, whereas pulmonary complications and wound infections were also noted in cases underwent T-tube repair (Table 3).

**Table 3:** Various factors on basis on the type of surgical technique among open surgical cases (n=34) ASA: American Society of Anesthesiologists; ERCP: endoscopic retrograde cholangiopancreatography; CBD: common bile duct; VAS: Visual Analog Score; SGOT: aspartate aminotransferase; SGPT: alanine aminotransferase

Different factors	Choledochoduodenostomy		Roux - en - y Hepaticojejumostomy		T-tube repair		P value
	Number (or) Mean	% or SD	Number of Mean	% or SD	Number of Mean	% or SD	
Age	55.93	10.28	55.58	10.33	51.75	9.29	0.6
Gender							
Female	10	71.43	8	66.67	3	37.50	0.2
Male	4	28.57	4	33.33	5	62.50	
ASA Class							
Class 2	10	71.43	9	75	4	50.00	0.4
Class 3	4	28.57	3	25	4	50.00	
Comorbidity							
Present	7	50.00	5	41.67	4	50.00	0.8
Absent	7	50.00	7	58.33	4	50.00	
Failed ERCP							
Yes	4	28.57	2	16.67	3	37.50	0.5
No	10	71.43	10	83.33	5	62.50	
Site of stone							
Ampulla	0	0	0	0	0	0	0.04
Distal CBD	5	35.71	5	41.67	1	12.50	
Mid- and distal CBD	1	7.14	4	33.33	0	0	
Mid-CBD	8	57.14	3	25.00	7	87.50	
CBD diameter	18.50	1.61	20.00	1.81	11.36	4.31	<0.001
Number of stones							
Multiple	12	85.71	8	66.67	5	62.50	0.3
Single	2	14.29	4	33.33	3	37.50	
Size of stones							
Small	4	28.57	3	25.00	8	100.00	0.007
Large	9	64.29	7	58.33	0	0	
Very Large	1	7.14	2	16.67	0	0	
Liver function tests							
Total serum Bilirubin	6.91	0.68	5.92	1.73	5.00	1.22	0.005
Direct Bilirubin	5.64	0.76	4.60	1.43	4.00	1.17	0.006
Alkaline Phosphatase	609.71	98.72	582.67	75.52	452.00	92.07	0.001
SGOT	44.14	15.96	46.17	16.21	43.00	11.31	0.6
SGPT	48.00	20.70	46.67	18.53	43.25	19.09	0.8
Operative time	167.14	22.68	215.00	22.76	167.50	16.69	<0.001
Intra operative complications	0	0	0	0	0	0	-
Postoperative complications							
Postoperative ICU requirement	2	14.29	0	0	0	0	0.2
Bile leak	1	7.14	2	16.67	0	0	0.4
Incisional hernia	0	0	2	16.67	0	0	0.1
Pulmonary complications	4	28.57	4	33.33	3	37.50	0.9
Recurrent stones	1	7.14	0	0	0	0	0.4
Wound infections	4	28.57	4	33.33	2	25.00	0.9
Stricture	1	7.14	0	0	0	0	0.4
Sump syndrome	0	0	-	-	-	-	-
Reflux gastritis	1	7.14	-	-	-	-	-
Mortality	0	0	0	0	0	0	-
NPO status	2.93	0.47	2.75	0.87	2.13	0.64	0.03
Duration of hospital stay	6.79	1.05	7.17	1.03	7.00	1.51	0.7
VAS at 24 hours	5.43	0.85	5.67	0.78	5.13	0.35	0.2
VAS at discharge	1.14	0.66	1.26	0.75	1.00	0.00	0.6
Time to return to work	18.00	3.06	19.83	1.75	18.50	2.73	0.2

## Discussion

The management of concurrent gallbladder and CBD stones is debated due to various treatment options. Before laparoscopic and endoscopic techniques emerged, open cholecystectomy and CBD exploration were the standard treatments for CBD stones. The introduction of laparoscopic cholecystectomy by Eric Muhe in 1989 revolutionized the treatment of gallbladder and biliary disorders, leading to quicker recovery, less postoperative pain, and shorter hospital stays [9].

In our study, the higher incidence of secondary bile duct stones compared to primary stones reflects the trend seen with gallstones, which are more common in older adults and women. Research by Grubnik et al. [10], Gad et al. [11], and Rogers et al. [12] also found that CBD stones primarily occur in middle-aged to elderly individuals, particularly among women.

The most common location for stones was the mid-CBD, followed by the distal CBD, with both mid- and distal CBD, and the ampulla. The average CBD diameter was 15.09±5.99 mm, mostly between 15 and 20 mm. 

Bansal et al. [13] reported similar success rates for single-stage and two-stage procedures (91.7% vs. 88.1%), while Rogers et al. [12] found no significant difference in total stone clearance rates (98% for ERCP/S vs. 88% for LCBDE). Our study also showed equal success rates, likely due to careful patient selection and a smaller sample size. Among the 11 (22%) patients, ERCP was followed by laparoscopic cholecystectomy within 24 to 48 hours, as longer intervals can increase morbidity.

In our study, in five patients who had laparoscopic exploration of the CBD, three (6%) cases underwent choledochoduodenostomy and two had T-tube repair. The average operative time was longer for the LCBDE group (328±19.24 minutes) than for the ERCP+LC group (149.09±17.58 minutes), as supported by Bansal et al. [13] and Gantois et al. [14]. The ERCP+LC group's time included the sum of individual procedure durations, as shown by Mohamed et al. [15]. The longer laparoscopic time in our study is due to technical challenges and the learning curve. Our results are consistent with studies showing slightly higher biliary fistula rates in the laparoscopic group and acute pancreatitis in the ERCP+LC group [14-16].

In our study, nearly all patients in the ERCP+LC group started oral feeding on the same day, while the laparoscopic CBDE group took an average of 2.6±1.52 days. The time to resume normal activities was also similar at 14.09 vs. 16.2 days. No mortality occurred in either group. During follow-up, one patient in the ERCP+LC group had an asymptomatic CBD stone recurrence after 15 months, detected during an unrelated ultrasound and removed via ERCP. Another patient in the laparoscopic group developed a stricture at the choledochoduodenostomy site, who also had a postoperative bile leak. These findings are consistent with Ding et al. [17] who reported higher rates of stone recurrence in the ERCP cohort, attributed to sphincterotomy compromising the sphincter of Oddi, leading to increased duodenobiliary reflux and higher CBD stone formation rates.

Open CBD exploration was performed in cases of multiple large stones, incomplete stone removal (failed ERCP), impacted distal CBD stones, significantly dilated CBD, distal CBD stricture, acute cholecystitis, and choledochoduodenal fistula. Among the nine (18%) patients with failed ERCP, six (12%) cases had acute cholangitis. The reasons for ERCP failures were unclear, as most patients were referred from other facilities, but likely stemmed from multiple or impacted stones and inadequate ERCP resources. Among the 34 (68%) open CBD exploration cases, 25 (73.5%) cases had multiple stones, while nine (26.47%) cases had a single stone. Specifically, 18 (52.94%) cases had mid-CBD calculi, 11 (32.35%) cases had distal CBD calculi, and five (14.70%) cases had both. 

In our research, open CBD exploration resulted in complete stone clearance for all failed ERCP cases and those with challenging stones, aligning with the findings of Kukar et al., who reported a 98% success rate through open CBD exploration [18]. In our study, the average duration of operation for the 34 (68%) cases treated with open CBD exploration was 184.12±31.15 minutes. Regarding immediate postoperative complications, three patients (8.82%) experienced non-significant bile leaks, which were managed conservatively. Additionally, 11 (32.35%) patients faced postoperative pulmonary issues (atelectasis in five (14.7%) patients, pleural effusion in three (8.82%) patients, pneumonia in three (8.82%) patients), which were treated with antibiotics and therapeutic pleural fluid aspiration as necessary. Furthermore, 10 (29.41%) patients developed superficial surgical site infections, which were conservatively managed through daily dressings.

The Kukar et al. [18] study noted Roux-en-Y hepaticojejunostomy as safe for most benign biliary issues. In our study, the operative duration was significantly longer in the HJ group (215±22.76 min) than in the CD (167.14±22.68 mins) and T-tube repair groups (167.50±16.69 min), mainly due to the extra time needed for the second anastomosis and Roux loop preparation in cases with intraabdominal adhesions. Surgical site infections occurred in four (28.57%) patients in the CD group, four (33.33%) in the HJ group, and three (25%) in the T-tube repair group, with no postoperative hemorrhage or mortality were reported. The average hospital stay was 6.79±1.05 days for the CD group, 7.17±1.03 days for the HJ group, and 7±1.51 days for the T-tube repair group. Pain scores at 24 hours post-operation and discharge, as well as the time to return to normal work, were similar across all three groups, indicating comparable short-term morbidity rates. In the CD group, 1 patient (7.14%) had recurrent stones after one year, and another one (7.14%) developed obstructive jaundice due to stricture at the anastomotic site after eight months. The stones were removed via ERCP, and the stricture was successfully dilated.

Ambreen et al. [19] reported no long-term morbidity in their T-tube repair cohort. Surgeons are often hesitant to perform CD due to the risk of sump syndrome, which can result from bile stasis and debris accumulation in the distal CBD after a side-to-side biliary-digestive anastomosis, potentially leading to cholangitis and hepatic abscesses. However, reported rates of sump syndrome are low, ranging from 0% to 8%, with our study showing a 0% incidence.

The possible reason includes a wide anastomosis in our study that allows effective drainage of food debris. The narrow lower portion of the anastomosis prevents food debris from entering and impacting the sump segment. Pre-operative ERCP was performed in 28.57% (4 out of 14) of the patients who underwent the procedure.

Strengths of the study

'Rendez-vous' technique combines the minimal access laparoscopy with intraoperative ERCP, offering several advantages such as it avoids unnecessary negative ERCPs despite improvements in MRI cholangiography. The gesture can be performed during a single general anaesthesia. Its effectiveness is similar to the two-step technique, allowing for simultaneous CBD exploration if needed. Although the operation takes longer, patient hospitalization is shorter than with the two-step method. The associated morbidity is lower.

Limitations

This study had a limited sample size, highlighting the need for a larger sample to minimize error margins. A cost-effectiveness analysis of the interventions was not performed, which greatly impacts intervention choices in developing countries. The median follow-up was 12 months; longer follow-up is crucial to assess long-term outcomes, including monitoring for recurrent stones, biliary strictures, and potential late-onset cholangiocarcinoma after bilioenteric drainage.

## Conclusions

The two-stage approach consisting of ERCP followed by laparoscopic cholecystectomy has proven to be the best in removal of CBD stones. Laparoscopic management of CBD stones requires expertise and has a steep learning curve. Open CBD exploration is an effective modality for treating complex cases of CBD stones, especially after the failure of ERCP but with higher short-term morbidity. Following open CBD exploration, all three biliary drainage procedures (CD, HJ or T-tube repair) have good outcomes. A large number of patients present to us with complex presentations of CBD stones; so open CBD exploration plays a pivotal role in their management at our institute. Thus, despite the rise of minimally invasive techniques, traditional open CBD exploration remains crucial and deserves recognition in our practice.

## References

[1] Sebghatollahi V, Parsa M, Minakari M, Azadbakht S (2023). A clinician's guide to gallstones and common bile duct (CBD): A study protocol for a systematic review and evidence-based recommendations. Health Sci Rep.

[2] Halldestam I, Enell EL, Kullman E, Borch K (2004). Development of symptoms and complications in individuals with asymptomatic gallstones. Br J Surg.

[3] Mohseni S, Bass GA, Forssten MP (2022). Common bile duct stones management: a network meta-analysis. J Trauma Acute Care Surg.

[4] Dimou FM, Adhikari D, Mehta HB, Riall TS (2016). Trends in follow-up of patients presenting to the emergency department with symptomatic cholelithiasis. J Am Coll Surg.

[5] Williams E, Beckingham I, El Sayed G, Gurusamy K, Sturgess R, Webster G, Young T (2017). Updated guideline on the management of common bile duct stones (CBDS). Gut.

[6] Vogt DP, Hermann RE (1981). Choledochoduodenostomy, choledochojejunostomy or sphincteroplasty for biliary and pancreatic disease. Ann Surg.

[7] Jarnagin W (2017). Blumgart’s Surgery of the Liver, Pancreas and Biliary Tract, Volume 1. Biliary Tract E.

[8] Winslow ER, Fialkowski EA, Linehan DC, Hawkins WG, Picus DD, Strasberg SM (2009). "Sideways": results of repair of biliary injuries using a policy of side-to-side hepatico-jejunostomy. Ann Surg.

[9] Barie PS, Franck P (2015). History of medical and surgical management of acute cholecystitis. Acute Cholecystitis.

[10] Grubnik VV, Tkachenko AI, Ilyashenko VV, Vorotyntseva KO (2012). Laparoscopic common bile duct exploration versus open surgery: comparative prospective randomized trial. Surg Endosc.

[11] Gad EH, Zakaria H, Kamel Y (2019). Surgical (open and laparoscopic) management of large difficult CBD stones after different sessions of endoscopic failure: a retrospective cohort study. Ann Med Surg (Lond).

[12] Rogers SJ, Cello JP, Horn JK (2010). Prospective randomized trial of LC+LCBDE vs ERCP/S+LC for common bile duct stone disease. Arch Surg.

[13] Bansal VK, Misra MC, Rajan K (2014). Single-stage laparoscopic common bile duct exploration and cholecystectomy versus two-stage endoscopic stone extraction followed by laparoscopic cholecystectomy for patients with concomitant gallbladder stones and common bile duct stones: a randomized controlled trial. Surg Endosc.

[14] Gantois D, Goudard Y, Bourgouin S, Pauleau G, de La Villéon B, Balandraud P (2020). One-stage laparoscopic procedure versus two-stage procedure in the management of common bile duct stones in patients aged 75 and more. J Visc Surg.

[15] Mohamed MA, Bahram MA, Ammar MS, Nassar AH (2015). One-session laparoscopic management of combined common bile duct and gallbladder stones versus sequential ERCP followed by laparoscopic cholecystectomy. J Laparoendosc Adv Surg Tech A.

[16] Noble H, Tranter S, Chesworth T, Norton S, Thompson M (2009). A randomized, clinical trial to compare endoscopic sphincterotomy and subsequent laparoscopic cholecystectomy with primary laparoscopic bile duct exploration during cholecystectomy in higher risk patients with choledocholithiasis. J Laparoendosc Adv Surg Tech A.

[17] Ding G, Cai W, Qin M (2014). Single-stage vs. two-stage management for concomitant gallstones and common bile duct stones: a prospective randomized trial with long-term follow-up. J Gastrointest Surg.

[18] Kukar M, Wilkinson N (2015). Surgical management of bile duct strictures. Indian J Surg.

[19] Ambreen M, Shaikh AR, Jamal A, Qureshi JN, Dalwani AG, Memon MM (2009). Primary closure versus T-tube drainage after open choledochotomy. Asian J Surg.

